# Infrared Spectroscopic Analysis in the Differentiation of Epithelial Misplacement From Adenocarcinoma in Sigmoid Colonic Adenomatous Polyps

**DOI:** 10.1177/2632010X221088960

**Published:** 2022-04-28

**Authors:** Jayakrupakar Nallala, Rebecca Griggs, Gavin R Lloyd, Nick Stone, Neil A Shepherd

**Affiliations:** 1 Biomedical Physics, School of Physics and Astronomy, University of Exeter, Exeter, UK; 2 Gloucestershire Cellular Pathology Laboratory, Cheltenham General Hospital, Cheltenham, Gloucestershire, UK; 3 Phenome Centre Birmingham, University of Birmingham, Birmingham, UK

**Keywords:** Epithelial misplacement, pseudo-invasion, adenocarcinoma, sigmoid colon, infra-red spectroscopy, spectral histopathology

## Abstract

**Purpose:**

The differential diagnosis of epithelial misplacement from invasive cancer in the colon is a challenging endeavour, augmented by the introduction of bowel cancer population screening. The main aim of the work is to test, as a proof-of concept study, the ability of the infrared spectroscopic imaging approach to differentiate epithelial misplacement from adenocarcinoma in sigmoid colonic adenomatous polyps.

**Methods:**

Ten samples from each of the four diagnostic groups, normal colonic mucosa, adenomatous polyps with low grade dysplasia, epithelial misplacement in adenomatous polyps and adenocarcinoma, were analysed using IR spectroscopic imaging and data processing methods. IR spectral images were subjected to data pre-processing and cluster analysis based segmentation to identify epithelial, connective tissue and stromal regions. Statistical analysis was carried out using principal component analysis and linear discriminant analysis based cross validation, to classify spectral features according to the pathology, and the diagnostic attributes were compared.

**Results:**

The combined 4-group classification model on an average showed a sensitivity of 64%, a specificity of 88% and an accuracy of 76% for prediction based on a ‘single spectrum’, whilst a ‘majority-vote’ prediction on an average showed a sensitivity of 73%, a specificity of 90% and an accuracy of 82%. The 2-group model, for the differential diagnosis of epithelial misplacement versus adenocarcinoma, showed an average sensitivity and specificity of 82.5% for prediction based on a ‘single spectrum’ whilst a ‘majority-vote’ classification showed an average sensitivity and specificity of 90%. A 92% area under the curve (AUC) value was obtained when evaluating the classifier using the Receiver Operating Characteristics (ROC) curves.

**Conclusions:**

IR spectroscopy shows promise in its ability to differentiate epithelial misplacement from adenocarcinoma in tissue sections, considered as one of the most challenging endeavours in population-wide diagnostic histopathology. Further studies with larger series, including cases with challenging diagnostic features are required to ascertain the capability of this modern digital pathology approach. In the long-term, IR spectroscopy based pathology which is relatively low-cost and rapid, could be a promising endeavour to consider for integration into the existing histopathology pathway, in particular for population based screening programmes where large number of samples are scrutinised.

## Introduction

Epithelial misplacement, also known as pseudo-invasion, is the phenomenon whereby epithelium, either non-neoplastic or adenomatous, is misplaced into the submucosa of the intestine, thereby mimicking malignancy. Although also seen in other polyps, such as serrated lesions and Peutz-Jeghers polyps, it is a particular feature of larger adenomatous polyps of the colon. First described in 1972,^1^ epithelial misplacement in adenomatous polyps provides particular diagnostic difficulties in its differentiation from malignancy.^2^

Epithelial misplacement is an especial feature in larger sigmoid colonic adenomatous polyps.^2^ Indeed, about 85% of all epithelial misplacement cases occur at this site. The sigmoid colon is the most mobile and narrowest part of the colon and the age at which large adenomatous polyps are most likely to occur means that many patients have associated diverticular disease. These factors ensure that large pedunculated polyps in the sigmoid colon are subject to mechanical and propulsive forces and thus polyps undergo traumatic changes and necrosis. These are thought to be the major determinants of epithelial misplacement and explain the propensity of the phenomenon for the sigmoid colon.^3^

The differential diagnosis, epithelial misplacement versus adenocarcinoma, is generally assessed by standard histological assessment. Factors such as the presence of associated lamina propria, haemosiderin deposition, mucin pools and a lack of a desmoplastic reaction, suggest epithelial misplacement and, more recently, accompaniment of the adenomatous epithelium by non-neoplastic epithelium, in the submucosa, is regarded as a helpful feature to indicate epithelial misplacement.^4^ Nonetheless, it is clear that the 2 conditions, epithelial misplacement and adenocarcinoma, can coexist in the same polyp, adding to the diagnostic difficulties.^3^

The diagnostic quandaries associated with epithelial misplacement have been especially exacerbated by the introduction of bowel cancer population screening. Such cases are selected into these programmes as these large adenomatous polyps bleed and the detection of occult blood is the screening method of choice. The diagnostic difficulties have been seen in many countries providing a population screening service. In the UK, the diagnostic difficulties have necessitated the establishment of an ‘Expert Board’ in which 3 specialised gastro-intestinal pathologists assess cases blind to ensure a consensus diagnosis.^3^ Even then, complete agreement is not necessarily achieved in every case. Other countries have introduced similar diagnostic boards because of the diagnostic difficulties.

If the differential diagnosis is so difficult using standard histopathological methodology, what of special techniques? Immunohistochemistry, using markers such as MMP-1, p53, E-cadherin and collagen IV, has been proposed as a useful technique^5^ but it is accepted that immunohistochemical methods only work well in classical cases and are not so helpful in equivocal cases.^6^ Other techniques, such as computerised 3-dimensional reconstruction, have not been found to add to the diagnostic armamentarium.^2^

So, there is an urgent requirement for additional technology to aid the diagnostic process in this difficult area. Modern approaches based on digital pathology that encompass biophotonics and data mining are a potential way forward to address this challenge. In particular, imaging approaches that can decipher the distinct tissue morphology and, at the same time, provide information on the tissue biomolecular composition should be valuable to help pathologists make objective decisions in difficult cases.

Infra-red (IR) spectroscopy, a label-free and non-destructive photonic approach, probes the vibrational bonds of biomolecules (such as DNA, proteins, lipids and sugars) and provides a spectral fingerprint directly representing the intrinsic biomolecular information of cells and tissues. Therefore any subtle changes within the biomolecular features can be subjected to investigation. In the field of digital pathology, IR spectroscopy has been used as a diagnostic tool for identifying molecular changes in different tissue types associated with benign and cancerous lesions.^7-14^ More recently, with rapid advances in instrumentation, speed of data collection and data analysis, the clinical applicability of IR spectroscopy has also been demonstrated.^15,16^ Therefore, given the diagnostic difficulties associated with epithelial misplacement in adenomatous polyps, it was hypothesised that IR imaging in combination with multivariate data analysis could differentiate epithelial misplacement from invasive cancer based on spectral fingerprints.

## Materials and Methods

### Samples

Ten tissue samples from each of normal colonic mucosa, adenomatous polyps, adenomatous polyps with epithelial misplacement and adenocarcinoma, collected as part of the Bowel Cancer Screening Programme (BCSP) for routine histopathological examination between 2010 and 2014 and stored as formalin-fixed paraffin-embedded (FFPE) blocks at the Gloucestershire Cellular Pathology Laboratory, were used in this study with approval of the Local Ethics Committee. The initial diagnosis was confirmed by 2 pathologists. From these tissue blocks, 3 consecutive 5 µm tissue sections were obtained. For each sample, the middle tissue section was placed on a calcium fluoride (CaF_2_) (Crystran, UK) substrate (a compatible substrate for IR measurements) and left unstained. The first and the third sections were floated on a standard glass slide for haematoxylin and eosin (H&E) staining as positive controls. This routine ensured that the regions of interest and the observed pathology were contiguous with the middle tissue sections used for IR imaging. The H&E sections were examined by an expert gastrointestinal pathologist (NAS) to confirm the initial diagnosis. The 40 sample group comprised 10 samples each from the 4 diagnostic groups (normal, adenomatous polyp with low grade dysplasia, adenomatous polyp with low grade dysplasia and epithelial misplacement and adenocarcinoma).

### Data collection

IR hyperspectral images were acquired from the unstained tissue sections using an FTIR imaging system (Agilent 620 FTIR microscope coupled to an Agilent 670 FTIR spectrometer) in the mid-IR spectral range covering 1000 to 3800 wavenumbers, at a spectral resolution of 4 cm^−1^ and averaged over 64 scans. For each image, mid-IR light from a Globar light source was focussed onto a sample spot of 140 × 140 µm^2^ (field of view constituting a single tile) through a 15 × cassegrain objective and a matched condenser. Using a motorised stage, several tiles were measured to cover the tissue region of interest. These regions were selected using HE image as reference on which the histopathological diagnosis was performed. To include as much as spectral/molecular heterogeneity, the diagnostic regions as well as their surrounding areas were measured. The number of tiles ranged from 1 × 1 tile (corresponds to ~140 × 140 µm^2^ of sample area) to 5 × 6 tiles (corresponds to ~700 µm × 844 µm of sample area).

Depending on the frequencies of the light absorbed by the sample, and the frequencies transmitted onto the imaging detector (128 × 128 pixel focal plane array-FPA), a hyperspectral image was generated. The hyperspectral image constituted the 2D image in x and y dimensions and spectra specific to the overall biomolecular information in the third dimension. For this study, high-definition spectral images were obtained at a pixel resolution of 1.1 × 1.1 µm^2^ (spatial detail) by using an enhanced magnification of ×36. For each sample, a tissue-free clear region of the substrate was measured as a background, using the same parameters as the sample, but averaged over 256 scans. The tissue sections were directly measured without any prior chemical dewaxing; instead a digital de-paraffinisation routine was used to neutralise paraffin signals and retain only the biomolecular information for analysis, as described previously.^17-20^ A total of 40 images were measured from the 40 individual samples constituting a dataset of 6 078 464 spectra at an average of 151 962 spectra per sample.

### Spectral data pre-processing

A preliminary spectral pre-processing was carried out to ensure only spectra of sufficient quality were used for statistical analysis. To this end, the IR spectra were initially truncated to the spectral fingerprint region of 1000 to 1800 cm^−1^ and then pre-processed using a modified extended multiplicative signal correction (EMSC) method for paraffin correction, baseline correction and spectra normalisation as described elsewhere.^17-20^ The pre-processed spectra were then subjected to multivariate analysis.

### Cluster analysis

In order to identify the regions of the tissue based on their specific spectral characteristics, K-means cluster analysis was carried out on each of the IR spectral images independently. K-means clustering is an iterative algorithm which partitions spectra into different classes (class numbers are pre-assigned) based on the spectral heterogeneity/similarity.^21,22^ Different class numbers were tested per image and per iteration to obtain the most representative cluster image. The labelled cluster images were then validated by an expert pathologist (NAS).

### Cross-validation by principal component analysis (PCA) and linear discriminant analysis (LDA)

IR spectra from the tissues, consisting of nuclear and cytoplasmic regions, identified using cluster analysis, were extracted to classify samples according to their diagnosis using PCA-LDA. The spectra were labelled with their respective pathology labels (normal, adenoma, epithelial misplacement and cancer) and subjected to PCA, to reduce the large dataset into smaller yet meaningful variables called principal components (PCs) or principal component loadings. The PC loadings were then subjected to analysis of variance (ANOVA) to select up to 25 PC loadings to demonstrate a significant difference between the pathology groups at the 95% significance level. The PC loadings retained after ANOVA were then used for pathology classification based on LDA cross-validation.

Leave-one-sample-out-cross-validation (LOSOCV) was carried out in which a single sample was taken out of the model and used as a test set with the remainder of the dataset used as the training set. The test sample was projected back onto the model and its predicted pathology was classified. This was carried out until each of the samples was independently tested. To make the prediction robust, the prediction was carried out using all spectra from the test samples.^23^ Alternatively, prediction based on a ‘majority voting’ was carried out. In this, instead of prediction using every spectrum from a test sample, a test sample showing more than 50% of its spectra assigned to the true class was considered a true positive.

## Results

**Figure 1.**
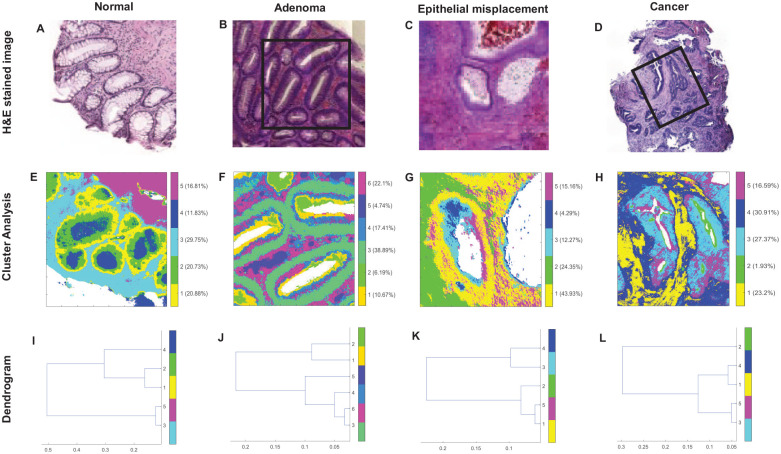
Histopathological segmentation of infrared spectral images based on cluster analysis and digital staining. Representative examples of digitally stained colon tissue sections from different pathologies (middle row): normal, adenoma, epithelial misplacement and cancer segmented into respective histological features, using H&E stained images (top row) as the ground truth. The corresponding dendrogram (bottom row) represents the heterogeneity of the clusters. The black boxes in B and D highlight the measured regions for F and H. The glandular epithelium is identified in 1E from cluster 1-nuclear region, cluster 2-cytoplasmic region, cluster 4-intracryptal mucus, and the connective tissue from cluster 3-lamina propria and cluster 5-submucosa. In Figure 1F which is adenomatous, the glandular epithelium is identified from cluster 3-nuclear region, clusters 1,2 and 4-cytoplasmic region, and the connective tissue from cluster 5. In the epithelial misplacement sample, the glandular regions can be identified in Figure 1G from clusters 1 and 5-nuclear region, cluster 3-cytoplasmic region, cluster 4-mucin within the duct and the connective tissue from cluster 2-submucosa. Finally, in Figure 1H which is a moderately differentiated adenocarcinoma, the glandular epithelium is identified from clusters 3 and 5-cancerous epithelium, and stroma from clusters 1 and 4. Scale bar=50 µm

The segmentation of spectral images using cluster analysis revealed the constituent histopathological features which could be morphologically compared to the reference H&E images (Figure 1A–D). For the normal (Figure 1E) and the adenomatous (Figure 1F) tissues, the features included glandular regions, lamina propria and adjacent connective tissue. The glandular regions were further demarcated into nuclear and cytoplasmic regions. For the epithelial misplacement tissue (Figure 1G), the characteristic histological features, including nuclear region, cytoplasmic region, submucosa and part of the cytoplasmic region representing mucus within the cytoplasm, were well demarcated. In the adenocarcinoma tissue (Figure 1H), a clear segmentation of the malignant epithelium and the surrounding stromal tissue was evident. The nuclear and the cytoplasmic regions were no longer distinguishable in the cancer tissue.

**Figure 2.**
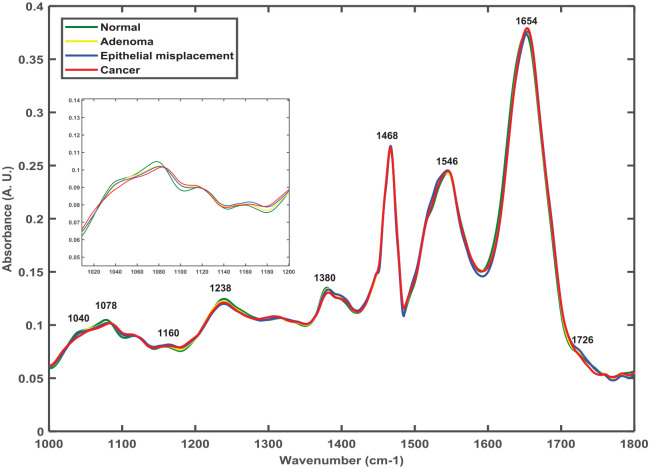
Mean spectra comparison of normal, adenoma, epithelial misplacement and cancer epithelium. Zoomed-in inset highlights spectral differences in spectral region 1000 to 1200 cm^−1^.

The pathology-wise comparison of mean spectra extracted from the epithelial regions of all the samples showed prominent IR spectral peaks at 1040 cm^−1^ (sugars), 1078 cm^−1^ (DNA), 1160 cm^−1^ (proteins), 1238 cm^−1^ (DNA), 1380 cm^−1^ (paraffin), 1468 cm^−1^ (paraffin), 1546 cm^−1^ (amide II of proteins), 1654 cm^−1^ (amide I of proteins) and 1726 cm^−1^ (phospholipids) (20) (Figure 2). This general comparison of the spectra highlighted only slight differences visually, with most of the differences evident in the 1000 to 1200 cm^−1^ spectral region (Figure 2-inset). The differences indicated spectral proximity between pathology groups and hence additional statistical analysis was needed for pathology-wise classification.

Pathology classification was performed using epithelial spectra (containing nuclear and cytoplasmic regions) of all the 4 groups as a ‘four-group classification model’, and between epithelial misplacement and cancer as a ‘two-group classification model’. For the 2-group classification model, in addition to using the combined nuclear and cytoplasmic regions constituting epithelial spectra, a nuclear-only spectral model was also tested.

**Figure 3.**
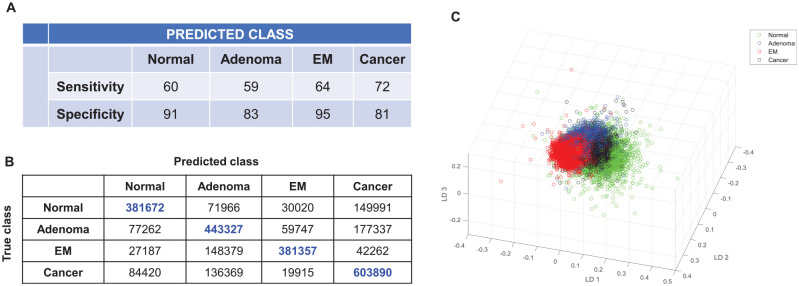
Classification attributes of normal, adenoma, epithelial misplacement and cancer pathology groups using leave-one-sample-out-cross-validation based on Principal Component Analysis followed by Linear Discriminant Analysis.(A) Sensitivity and specificity values for the 4 pathology groups (B) Confusion matrix corresponding to the 4-pathology group model and (C). Scatter plot for the 4 pathology groups generated from the first 3 linear discriminant scores

For the 4-group model (Figure 3A), an average sensitivity of 64%, an average specificity of 88% and an accuracy of 76% was observed, with the cancer group showing the highest sensitivity of 72% and the epithelial misplacement group showing a sensitivity of 64%. In terms of specificity, epithelial misplacement showed the highest at 95%. These values indicated partial overlap of spectra across groups which could be deduced from the confusion matrix (Figure 3B). Based on the confusion matrix, the majority of the overlap for the normal and the adenoma groups was with the cancer group at 24% and 23% respectively, while the majority of the overlap for the epithelial misplacement was with the adenoma group at 25%.

The separation between the groups could be comprehended using the scatter plot (Figure 3C) generated using the most discriminatory linear discriminant (LD) scores. Alternatively, using the ‘majority voting’ classification, an improvement in the overall classification attributes was observed in comparison to the single spectrum prediction, for which an average sensitivity of 73%, average specificity of 90% and an accuracy of 82% was obtained (Supplemental Information 1).

**Figure 4.**
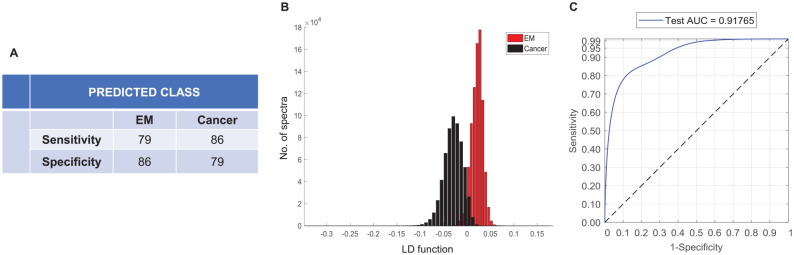
Classification attributes of epithelial misplacement versus cancer using leave one sample out cross validation based on Principal Component Analysis followed by Linear Discriminant Analysis.(A) Sensitivity and specificity for epithelial misplacement and cancer groups. (B) Histograms showing the linear discriminant separation between epithelial misplacement and cancer groups. (C) Receiver operating characteristic (ROC) curves representing the classification performance

The 2-group classification model specific for epithelial misplacement and cancer showed an improved average sensitivity and specificity of 82.5% (Figure 4A). The separation was more prominent when visualised using the linear discriminant plot in comparison to the 4-group model (Figure 4B). The classifier performance evaluated using the Receiver Operating Characteristics (ROC) curves showed an area under the curve (AUC) value of 92% (Figure 4C). Alternatively, using the ‘majority-vote’ classification, a further improvement in sensitivity and specificity to 90% in both groups was observed (Supplemental Information 2).

**Figure 5.**
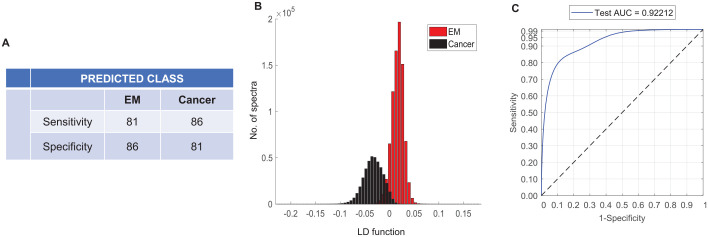
Classification attributes of epithelial misplacement versus cancer based on nuclear-only spectral features using leave one sample out cross validation based on Principal Component Analysis followed by Linear Discriminant Analysis. (A) Sensitivity and specificity for epithelial misplacement and cancer groups. (B) Histograms showing the linear discriminant separation between epithelial misplacement and cancer groups. (C) Receiver operating characteristic (ROC) curves representing the classification performance.

The 2-group model using the ‘nuclear-only’ region of the epithelial misplacement versus cancer showed only a slight improvement with an average sensitivity and specificity of 83.5% (Figure 5A) (compared to 82.5% for combined nuclear and cytoplasmic model). A similar trend for separation on the linear discriminant plot (Figure 5B), and the AUC performance of 92% was observed (Figure 5C). Finally, the 2-group nuclear-only model based on majority-vote classification showed a similar outcome as that of the epithelium-based classification with a sensitivity and a specificity of 90% (data not shown).

## Discussion

Whilst the differential diagnosis of epithelial misplacement and adenocarcinoma, in an adenomatous polyp, has been a major diagnostic conundrum for pathologists for years,^1^ the introduction of population-based colorectal cancer screening has greatly exacerbated the problems and this has been recognised in the UK for 15 years now, necessitating the establishment of a specific diagnostic board to address, nationally, this diagnostic quandary.^2^ Adjunctive tests, such as immunohistochemistry, usually so useful in the diagnostic pathologist’s armamentarium, have not proven useful so far in this difficult area so there is a crying need for a reliable adjunctive method that can accurately differentiate these 2 conditions.

In this novel endeavour, the feasibility of a biophotonic approach based on IR spectroscopic imaging has been used to address the differential diagnosis of epithelial misplacement from invasive cancer. The employment of digital data processing and multivariate statistical analyses to spectral imaging data provided image patterns comparable to that of conventional histopathology for the recognition of various histological structures. These included features such as normal epithelium, connective tissue, mucin signatures for benign entities and malignant epithelium and stroma for invasive cancer. The use of high-definition IR imaging enabled further delineation of histological features into much smaller epithelial constituents, such as nuclear region, cytoplasmic region and intra-cytoplasmic mucus, etc. The distinct connective tissue features were also identified as lamina propria, submucosa and stroma across all the pathology groups. The patterns associated with the pathological progression from normal to adenomatous to cancerous stages, usually involving a gradual loss of tissue architecture and reduction in mucus secretion in the intra-cytoplasmic region, have been identified. With the added benefit of capturing molecular information from IR spectra, in concert with spatial information, the spectral features were used for pathology classification.

A high AUC value of 92% (and an average sensitivity and specificity of 83%), for discriminating epithelial misplacement from invasive cancer, based on epithelial signatures (from both nuclear and cytoplasmic regions or nuclear-only region) demonstrated that this novel digital approach could be useful for diagnostic purposes. The overall sensitivity decreased to 64% when using the combined 4-group model. This might be expected, considering that there are 3 non-malignant groups i.e. normal, adenoma and epithelial misplacement, which may show molecular homogeneity as a part of the same model.

The confusion matrix provided further insights into the aforementioned molecular homogeneity or overlap. For example, a 24% overlap of normal spectra with the cancer group, as observed here, was also reported previously, including a study involving more than 100 colon samples. It has been postulated that, in colon the normal epithelial cells are highly proliferative and actively dividing, a property similar to neoplastic cells with high mitotic rates. Therefore, between such highly proliferative cells and tumours that are moderately differentiated, the net spectral variance is only slightly increased.^22,24^ It is however less surprising to see an overlap of the adenoma spectra with the cancer group suggesting some of the early molecular changes indicative of cancer are being detected by the model as observed previously.^24^ It is known that adenomas accumulate molecular changes pertaining to increased mitotic rate and do not always show phenotypic manifestations in the early stages of neoplastic transformation. The overlap of the epithelial misplacement spectra with the adenoma group was, however, expected because epithelial misplacement is a particular feature of large adenomatous polyps and some of the adenomatous signatures could be shared between these groups.

It is important to note that the prediction threshold was set in a stringent manner where each spectrum from the test sample was tested against the training set consisting of about 3 million spectra. Such a stringent model tends to have increased overlap between spectral groups.

In comparison, the relatively less stringent ‘majority-vote’ classification improved the prediction attributes to an average sensitivity of 73% for the 4-group model. In a similar trend, for the 2-group epithelial misplacement and invasive group model, the majority-vote classification improved the sensitivity and specificity values to 90% each. In general, spectral overlaps observed in multi-group models, such as this, could be reduced by having a training set involving large patient numbers. In this proof-of-concept study, limited patients per group were included to test the feasibility of the approach. In addition, other advanced machine learning algorithms such as Random Forests, Support Vector Machines, Artificial Neural Networks etc., might reduce spectral overlap between groups and provide improved diagnostic attributes, although they remain to be tested.

Additional analysis was also performed taking into consideration the desmoplastic response surrounding carcinoma versus epithelial misplacement without any desmoplastic response, to see how this impacts the classification results. Two separate models involving (i) epithelial misplacement versus cancer and its surrounding stroma, and (ii) epithelial misplacement and its surrounding connective tissue versus cancer and its surrounding stroma were tested. Both these models did not show any improvement in the classification attributes, and the results were comparable (data not shown) to the epithelial-only classification attributes presented in the results. Although consideration of desmoplastic response is an important factor histopathologically, it appears that the spectral attributes involving only the epithelial features are sufficient to distinguish between epithelial misplacement and invasive cancer.

Nonetheless, the observed classification attributes demonstrate the capability of the model to delineate subtle differences between the groups. For example, the epithelial misplacement samples might be harbouring subtle differences because of their surrounding milieu, in comparison to the normal or the adenoma samples. The differences could also be attributed to the molecular signatures of the mucus pools produced in epithelial misplacement, which are confined to the submucosa, unlike the mucus produced in the normal epithelium that is secreted into the lumen of the colon.^4^ This demonstrates that epithelial misplacement samples have spectral profiles that are distinct from the other non-neoplastic groups. Although this needs further investigation, the classical features of epithelial misplacement involving the combination of higher mucinous content and haemosiderin deposition, as well as its surrounding connective tissue milieu, could be the defining factors of their distinct profile.

Based on these observations, together with the high sensitivity of the model to distinguish epithelial misplacement from invasive cancer, the current approach using IR spectroscopic imaging appears to be a promising endeavour to pursue. In the future, IR spectral-histopathology could have a beneficial health economic impact, considering cost and time-effectiveness in the decision making of difficult cases such as epithelial misplacement from invasive cancer.

For comparison, a standard histopathology procedure necessitates staining the tissue section for examination, while IR spectroscopy can be performed directly on unstained tissue sections, thereby saving on cost and time. In addition to an IR instrument and a standard computational cost, the only other requisite would be the need to use specialised substrates, such as CaF_2_ in this case, to deposit the samples. However, the cost for a specialised substrate would balance out with the cost involved in H&E staining procedure or the need for multiple HE stained tissues or immunochemical stains to confer a diagnosis, while IR spectroscopy can provide the molecular information from a single unstained section.

From time implications point of view, using the current IR imaging system, a sample of 390 × 380 µm^2^ (average size of the samples measured in this study) takes ~1 hour measurement time with an additional 10 minutes for analysing the sample (pre-processing and cluster analysis). It is important to reiterate that, images for this study were obtained in a ‘high-definition’ modality (1.1 × 1.1 µm^2^ pixel size). In comparison, a ‘standard definition’ image (5.5 × 5.5 µm^2^ pixel size) of a similar size would take ~20 minutes measurement time. The pathology of the sample could be then automatically predicted in few minutes by projecting it onto already modelled data bases. Other advanced technologies that use mid-IR laser based discrete frequency imaging such as Quantum Cascade^7,8,16^ and/or Supercontinuum lasers,^25^ have faster measurement times and use parameterised data points which can significantly reduce both measurement and analytical time.

In addition, it could be possible to automate spectral histopathology to run samples in continuity, which would be beneficial over a manually driven pathology routine. This could reduce workload in hospitals especially in a population wide screening programmes such as bowel cancer screening. Furthermore, the automation capability also means that decision making of difficult cases can be provided by non-specialists in specific pathologies. This is particularly useful where there is limited availability of specialist pathologists needed for diagnosing difficult cases such as in the differential diagnosis of epithelial misplacement from invasive cancer.

The current work is a proof-of-concept study covering a small sample set specifically to address the issue of differential diagnosis of epithelial misplacement from invasive cancer. It might be expected that larger studies would ascertain the practical use of IR spectroscopic imaging as a clinical translation tool.

It is accepted that this initial study has been undertaken on classical cases of epithelial misplacement and adenocarcinoma. So it could be that, like with immunohistochemical studies,^5^ this technique may be less discriminatory in difficult and challenging cases. Nevertheless further larger studies will clarify this issue. This modern digital pathology approach, with the scope for automation driven by artificial intelligence and portability, could be developed as a pathology tool that could reduce the overall workload, especially in screening samples, and shows promise in aiding the considerable problems associated with the ‘diagnostic conundrum of the century’ as described by one of our authors.^6^

## Supplemental Material

sj-pdf-1-pat-10.1177_2632010X221088960 – Supplemental material for Infrared Spectroscopic Analysis in the Differentiation of Epithelial Misplacement From Adenocarcinoma in Sigmoid Colonic Adenomatous Polyps Click here for additional data file. Supplemental material, sj-pdf-1-pat-10.1177_2632010X221088960 for Infrared Spectroscopic Analysis in the Differentiation of Epithelial Misplacement From Adenocarcinoma in Sigmoid Colonic Adenomatous Polyps by Jayakrupakar Nallala, Rebecca Griggs, Gavin R Lloyd, Nick Stone and Neil A Shepherd in Clinical Pathology

sj-pdf-2-pat-10.1177_2632010X221088960 – Supplemental material for Infrared Spectroscopic Analysis in the Differentiation of Epithelial Misplacement From Adenocarcinoma in Sigmoid Colonic Adenomatous Polyps Click here for additional data file. Supplemental material, sj-pdf-2-pat-10.1177_2632010X221088960 for Infrared Spectroscopic Analysis in the Differentiation of Epithelial Misplacement From Adenocarcinoma in Sigmoid Colonic Adenomatous Polyps by Jayakrupakar Nallala, Rebecca Griggs, Gavin R Lloyd, Nick Stone and Neil A Shepherd in Clinical Pathology
