# Supplementary material for: Infrared Spectroscopic Analysis in the Differentiation of Epithelial Misplacement From Adenocarcinoma in Sigmoid Colonic Adenomatous Polyps
Source: Clin Pathol. 2022 Apr 28;15:2632010X221088960. doi: 10.1177/2632010X221088960 (PMC9058331; doi:10.1177/2632010X221088960)
Supplement: sj-pdf-1-pat-10.1177_2632010X221088960 – Supplemental material for Infrared Spectroscopic Analysis in the Differentiation of Epithelial Misplacement From Adenocarcinoma in Sigmoid Colonic Adenomatous Polyps [file sj-pdf-1-pat-10.1177_2632010X221088960.pdf]

A

	PREDICTED CLASS			
	Normal	Adenoma	EM	Cancer
Sensitivity	90	60	70	70
Specificity	91	82	100	85

B

Predicted class

True class	Normal	Adenoma	EM	Cancer
**Normal**	**9**	0	0	1
**Adenoma**	1	**6**	0	3
**EM**	0	3	**7**	0
**Cancer**	1	2	0	**7**
