# Supplementary material for: Infrared Spectroscopic Analysis in the Differentiation of Epithelial Misplacement From Adenocarcinoma in Sigmoid Colonic Adenomatous Polyps
Source: Clin Pathol. 2022 Apr 28;15:2632010X221088960. doi: 10.1177/2632010X221088960 (PMC9058331; doi:10.1177/2632010X221088960)
Supplement: sj-pdf-2-pat-10.1177_2632010X221088960 – Supplemental material for Infrared Spectroscopic Analysis in the Differentiation of Epithelial Misplacement From Adenocarcinoma in Sigmoid Colonic Adenomatous Polyps [file sj-pdf-2-pat-10.1177_2632010X221088960.pdf]

A

	PREDICTED CLASS		
		**EM**	**Cancer**
	**Sensitivity**	90	90
	**Specificity**	90	90

B

Predicted class

True class		**EM**	**Cancer**
	**EM**	9	1
	**Cancer**	1	9
